# Tumor ratio of unsaturated to saturated sulfatide species is associated with disease-free survival in intrahepatic cholangiocarcinoma

**DOI:** 10.1007/s13402-022-00766-6

**Published:** 2023-01-11

**Authors:** Lennart Huizing, Lin Chen, Anjali A. Roeth, Lara R. Heij, Bryn Flinders, Stefan A. W. Bouwense, Benjamin Balluff, Ulf P. Neumann, Ron M. A. Heeren, Steven W. M. Olde Damink, Rob J. Vreeken, Frank G. Schaap

**Affiliations:** 1 Maastricht Multimodal Molecular Imaging Institute (M4I), Maastricht University, Universiteitssingel 50, 6229 ER Maastricht, The Netherlands; 2 Department of Surgery, Maastricht University Medical Center and NUTRIM School of Nutrition and Translational Research in Metabolism, Maastricht University, PO BOX 616, 6200 MD Maastricht, The Netherlands; 3 Department of General, Visceral and Transplantation Surgery, RWTH University Hospital Aachen, Aachen, Germany; 4 Janssen Research & Development, Turnhoutseweg 30, 2340 Beerse, Belgium

**Keywords:** Mass spectrometry imaging, Glycosphingolipids, Cholangiocarcinoma, Colorectal liver metastasis, Hepatocellular carcinoma, Disease-free survival

## Abstract

**Purpose:**

Cholangiocarcinoma (CCA) is a malignancy arising from the bile duct epithelium and has a poor outcome. Sulfatides are lipid components of lipid rafts, and are implicated in several cancer types. In the liver, sulfatides are specifically present in the bile ducts. Here, sulfatide abundance and composition were analyzed using mass spectrometry imaging in intrahepatic CCA (iCCA) tumor tissue, and correlated with tumor biology and clinical outcomes.

**Methods:**

Sulfatides were analyzed in iCCA (n = 17), hepatocellular carcinoma (HCC, n = 10) and colorectal liver metastasis (CRLM, n = 10) tumor samples, as well as tumor-distal samples (control, n = 16) using mass spectrometry imaging. Levels of sulfatides as well as the relative amount in structural classes were compared between groups, and were correlated with clinical outcomes for iCCA patients.

**Results:**

Sulfatide localization was limited to the respective tumor areas and the bile ducts. Sulfatide abundance was similar in iCCA and control tissue, while intensities were notably higher in CRLM in comparison with control (18-fold, *P* < 0.05) and HCC tissue (47-fold, *P* < 0.001). Considerable variation in sulfatide abundance was observed in iCCA tumors. A high ratio of unsaturated to saturated sulfatides was associated with reduced disease-free survival (10 vs. 20 months) in iCCA. The sulfatide pattern in HCC deviated from the other groups, with a higher relative abundance of odd- versus even-chain sulfatides.

**Conclusion:**

Sulfatides were found in tumor tissue of patients with iCCA, with sulfatide abundance per pixel being similar to bile ducts. In this explorative study, sulfatide abundance was not related to overall survival of iCCA patients. A high ratio of unsaturated to saturated sulfatides was associated with earlier tumor recurrence in patients with iCCA.

**Supplementary Information:**

The online version contains supplementary material available at 10.1007/s13402-022-00766-6.

## Introduction

Cholangiocarcinoma (CCA) is a malignancy arising from the epithelium of the biliary tree. CCA is classified based on the anatomical origin as intrahepatic, perihilar or extrahepatic. Owing to late diagnosis, frequently in a stadium where metastasis has already occurred, and with limited or no treatment options, patients with CCA have a poor prognosis. Surgery is the only curative option for the minority of patients with a resectable tumor, and typically involves extensive procedures that are accompanied by considerable post-operative morbidity and mortality. Chemoresistance contributes to the low success rate of systemic chemotherapy in palliative treatment of CCA patients with unresectable tumors, metastatic spread, or those otherwise ineligible for surgery [1, 2].

We recently identified sulfatides as a class of lipids that -within the liver- are uniquely present in the bile ducts [3]. Sulfatides are derived by galactosylation of ceramide and subsequent 3’-sulfation of the carbohydrate moiety (Supplemental Fig. 1) [4]. Individual sulfatide species show structural variation in the *N*-acyl chain of ceramide, with alterations in length, occurrence and number of unsaturated bonds, and presence of a hydroxyl group at C2. The function of these negatively charged sulfoglycosphingolipids in the liver is not known. In general, sulfatides are plasma membrane lipids present in the exoleaflet that act as ligand for several proteins, *e.g.* P-selectin, thereby modulating cell–cell interaction and cellular processes [4–6]. Observation of sulfatide-devoid bile ducts and extraductular presence of (atypical) bile salts in the liver parenchyma in patients with advanced primary sclerosing cholangitis, led us to propose that sulfatides protect cholangiocytes from bile salt toxicity [3]. Primary sclerosing cholangitis is a risk factor for development of CCA [7].

Ceramides are pro-apoptotic molecules that can induce cell death [8]. Elevated ceramide content in breast cancer has been associated with a less aggressive tumor phenotype, indicating functionality of routes leading to programmed cell death [9]. Shifting the balance from apoptotic to pro-survival signaling is a mechanism that contributes to chemoresistance of tumor cells [10, 11]. It is currently unexplored if utilization of ceramide for sulfatide synthesis impacts on cancer cell behavior. Levels of sulfatides are in general elevated in renal cell carcinoma [6, 12–15], and this has been mechanistically linked with upregulation of sulfatide synthetic genes by HIF1α and inactivating mutations in the pathway controlling degradation of this transcription factor [12].

Sulfatides have not been studied previously in the context of CCA. In fluke-associated CCA, accumulation of lactosylceramides (LacCer), the products of another ceramide-metabolizing route that potentially alters the balance between pro- and anti-apoptotic signaling, has been reported. Notably, the ratio between a particular 2-hydroxylated and non-hydroxylated LacCer species (viz*.* LacCer d18:1-h23:0) was associated with a pronounced reduction in survival of patients [16]. Since sulfatides have been implicated in various types of cancer, we sought to determine whether sulfatides are expressed in tumors originating from the bile duct. Using mass spectrometry imaging (MSI) we studied the spatial distribution of sulfatides in intrahepatic CCA (iCCA) tissue and compared the abundance and composition of sulfatides in iCCA with liver tumors originating from parenchymal cells or the colon, or tumor-distal control tissue. Correlations with clinical outcomes and tumor biology were evaluated to test the hypothesis that specific sulfatide features are associated with poor outcomes in iCCA.

## Materials and methods

### Chemicals

All solvents for MSI (methanol, chloroform, ethanol, water) were HPLC grade or better, and were obtained from Biosolve bv. (Valkenswaard, The Netherlands). Norharmane was obtained from Sigma-Aldrich (Zwijndrecht, The Netherlands).

### Patients

Patient samples were procured in the framework of prospective local biobank programs that were initiated in 2017 (Maastricht) and 2009 (Aachen). For MSI, the workflow for cryopreservation of tissues was optimized to minimize time between surgical excision and diagnostic processing of specimens by the pathologist. The biobank programs were approved by local ethical committees (Maastricht #16–4-153, Aachen EK 206/09), and all patients scheduled for surgical treatment of hepatobiliary tumors were eligible for participation. Participating patients provided written informed consent for storage and research use of their specimens.

MSI was used to study the occurrence, and spatial distribution of sulfatides in control and liver tumor tissue. Specimens were obtained from patients scheduled for curative-intent surgery for intrahepatic cholangiocarcinoma (iCCA, n = 17), hepatocellular carcinoma (HCC, n = 10) or colorectal liver metastasis (CRLM, n = 10). All initial diagnoses were confirmed by pathological evaluation of resection specimens. Characteristics of the iCCA group are reported in Supplemental Table 1. Paired tumor-distal tissue with gross normal appearance was gathered from patients with HCC or CRLM, and considered as control tissue (n = 8 per group, total n = 16). Tumor-distal tissue was not available for patients with iCCA due to the nature of the resection (non-anatomical, parenchyma-sparing) in this patient group. Following excision, tissues were frozen within one hour in liquid nitrogen and subsequently stored at -80 °C. A second tumor sample from a different part of the tumor, was obtained in 6 cases of iCCA and used to study intratumor heterogeneity.

### MALDI MSI analysis of liver tissues

Fresh-frozen liver tissues were cut using a cryomicrotome at -18 °C. For MSI, 10 µm sections were cut and mounted on indium-tin-oxide coated conductive glass slides (Delta Technologies, Loveland, CO, USA). For immunohistochemical staining, consecutive sections of 6 µm were prepared on poly-lysine coated glass slides (Delta Technologies, Loveland, CO, USA). All glass slides were stored at -80 °C until analysis.

For MSI, glass slides transferred from -80 °C were first desiccated in a vacuum desiccator for 30 min to prevent water condensation and subsequent delocalization of molecules. To check for consistent ionization conditions/mass spectrometric performance, one µl of a sulfatide mixture (Sigma-Aldrich Zwijndrecht, The Netherlands; catalog# 131305P) was spotted next to each tissue section in a concentration range of 1–50 µg/mL (unspiked) or 5–100 µg/mL when spiked in a human liver homogenate. These calibration standards were used to set up the mass spectrometers to obtain equal signal between measurements. A solution of norharmane (7.0 mg/mL in 2:1 CHCl_3_:methanol) was sprayed (flow rate: 0.12 mL/min, velocity: 1200 mm/min, track spacing: 3 mm, temperature: 30 °C) onto the slide using a TM-sprayer (HTX technologies, Chapel Hill, North Carolina, USA), with a total of 15 layers.

For high-throughput screening the entire tissue section was imaged on a Rapiflex TissueTyper (Bruker Daltonik GmbH, Bremen, Germany) at 50 × 50 µm pixel size in dual polarity mode, thus part of the pixel area was analyzed in positive ion mode and part in negative ion mode as described previously [17]. Briefly, the laser was rastered over a 25 by 25 µm area first in negative ion mode, and the run was repeated in positive ion mode in a consecutive run with a 25 by 25 µm offset to provide molecular information in the same 50 × 50 µm square.

For both ion modes the mass range was set to 300–2000 m/z measured in reflectron mode with a digitizer rate of 2.5 GS/s. The dual polarity approach was chosen to simultaneously detect the immediate precursor of sulfatides, i.e. βGalCer in the positive ion mode [17]. It turned out that sensitivity was not sufficient to reliably detect this compound, and positive ion mode data was not further analyzed for this study.

Based on the Rapiflex MSI data and the histological (H&E staining) information, a region of interest was selected on consecutive sections for high-resolution mass measurements at higher spatial resolution (20 × 20 µm pixel size) on a Solarix XR Fourier-Transform ion cyclotron resonance (FT-ICR) mass spectrometer (Bruker Daltonik GmbH, Bremen, Germany) coupled to a 9.4 T superconducting magnet. An area of approximately 10,000 pixels was selected per sample. The mass range was set to 300–2000 m*/z* with a 1.101 s transient length (*i.e.* the duration of time that ions were analyzed in the ion cyclotron resonance cell), which resulted in a mass resolution of approximately 140,000 at *m/z* 800. Laser focus was set to minimum and smart walk turned off.

#### Immunohistochemistry

Imaged sections were stained with haematoxylin and eosin (H&E, Merck KGaA, Darmstadt, Germany) to allow the overlay of molecular and histological information. Prior to staining, matrix was washed away by submersing the slides in methanol for 3 min, followed by rehydration in water for 2 min.

Bile ducts were identified by cytokeratin 19 staining. For this, 5 µm thick sections adjacent to sections used for MSI, were air-dried for 15 min, fixed in acetone for 10 min, and air-dried for 30 min at room temperature. Next, sections underwent blocking of endogenous peroxidase activity in methanol/0.6% H_2_O_2_ for 30 min and subsequently incubated in blocking solution (5% bovine serum albumin in PBS) for 30 min. Sections were incubated with a polyclonal antibody against human cytokeratin 19 (R&D Systems, catalog# AF3056), and peroxidase-labelled secondary antibody (DAKO GmbH, Jena Germany) for 1 h at room temperature. Liquid DAB + Substrate Chromogen System (DAKO) was used as peroxidase substrate. Haematoxylin was used as counterstain.

#### Analysis of MSI data

Annotations of the histological findings (H&E) were drawn in QuPath (Version 0.1.2, The Queen's University of Belfast, Northern Ireland) in collaboration with an experienced pathologist (LRH). Annotations were exported to Scils lab (Version 2019c Premium 3D, Bruker Daltonik GmbH, Bremen, Germany) using an in-house written Matlab® script (version r2019a).

Comparison of sulfatide abundance and composition between groups was performed in Scils Lab. Signals were root mean square normalized to the overall signal to ensure optimal pixel-to-pixel and sample-to-sample comparison [18]. A mass list of 126 theoretical *m/*z values for both non-hydroxylated and hydroxylated sulfatides with a total carbon chain length of 30–50 containing 0–2 unsaturations in the *N*-acyl chain, was created and compared to signals present in the tissue. A mass error up to 2 ppm was tolerated, and identities of abundant sulfatides were further confirmed by MS/MS. Note that the employed MS approach cannot discriminate between sulfatides containing the sphingoid base that dominates in humans (*i.e.* sphingosine d18:1) and a saturated *N*-acyl chain, and species comprising the minor sphingoid base dihydrosphingosine (d18:0) and an unsaturated *N*-acyl chain. For instance, ST40:1 can be either d18:1/22:0 or d18:0/22:1. A list of detected sulfatides is provided in Supplemental Tables 2 and 3.

Sulfatide intensities within the tumor region (iCCA, CRLM, HCC) or bile duct in case of tumor-distal control tissue, were exported from Scils lab as maximum mean intensity, *i.e.* for each region a mean spectrum, based on the pixels in that region, was obtained for each *m/z* interval in the spectrum corresponding to a particular sulfatide. The maximum intensity within that interval was exported to Excel/GraphPad Prism for statistical analysis. Maximum peak height rather than area under the curve (AUC) was chosen for analysis of FT-ICR data since the line shape in FT-ICR is affected by multiple factors next to ion count, such as *e.g.* pressure, ions in the cell etc., and the AUC therefore does not accurately represent signal intensity. This is in contrast to time-of-flight based instruments, where the peaks are fit to ions reaching the detector [19, 20].

Total sulfatide intensity per sample was calculated as summed intensities of all detected sulfatide species. Additionally, for each sample summed intensities were calculated for non-hydroxylated, hydroxylated, saturated, unsaturated, even chain and odd chain species, to arrive at totals for the respective sulfatide classes of interest. From the respective totals, ratios were calculated for hydroxylated/non-hydroxylated, unsaturated/saturated and even/odd-chain species for each sample.

A hierarchical clustering (Euclidean distance, average linkage) of the samples based on the intensities of constituent sulfatides was performed and a heatmap was created using R.

### Statistical analysis

Between-group differences were tested using non-parametric ANOVA, with post-hoc testing if overall *P* value was below 0.05. Dunn’s multiple comparison test was used to compare total sulfatide signal between all pairs of groups. For other sulfatide features, only differences versus the control group were evaluated using Dunn’s test.

Disease-free and overall survival analysis were performed for sulfatide-related parameters, initially by Cox proportional hazard analysis. For all outcomes with a *P* value smaller than 0.10, Kaplan–Meier plots were created based on a dynamic threshold for every molecular feature at the 50% quantile, ensuring a split of the patients into equally sized groups.

For the iCCA group, tumor features (TNM stage, tumor grade) were correlated with sulfatide-related parameters (*i.e.* signal intensity, hydroxylation ratio, and unsaturation index). Correlations were evaluated in R (Version 3.6.2, 2019) using a Wilcoxon signed-rank test, and *P* values were adjusted for multiple testing using Benjamini-Hochberg.

Data are reported as median [interquartile range]. *P* values < 0.05 were considered statistically significant. Statistical analysis were conducted in R and GraphPad Prism 9.0 (GraphPad Software LLC).

## Results

### Sulfatides are present in iCCA tumor tissue

**Fig. 1:**
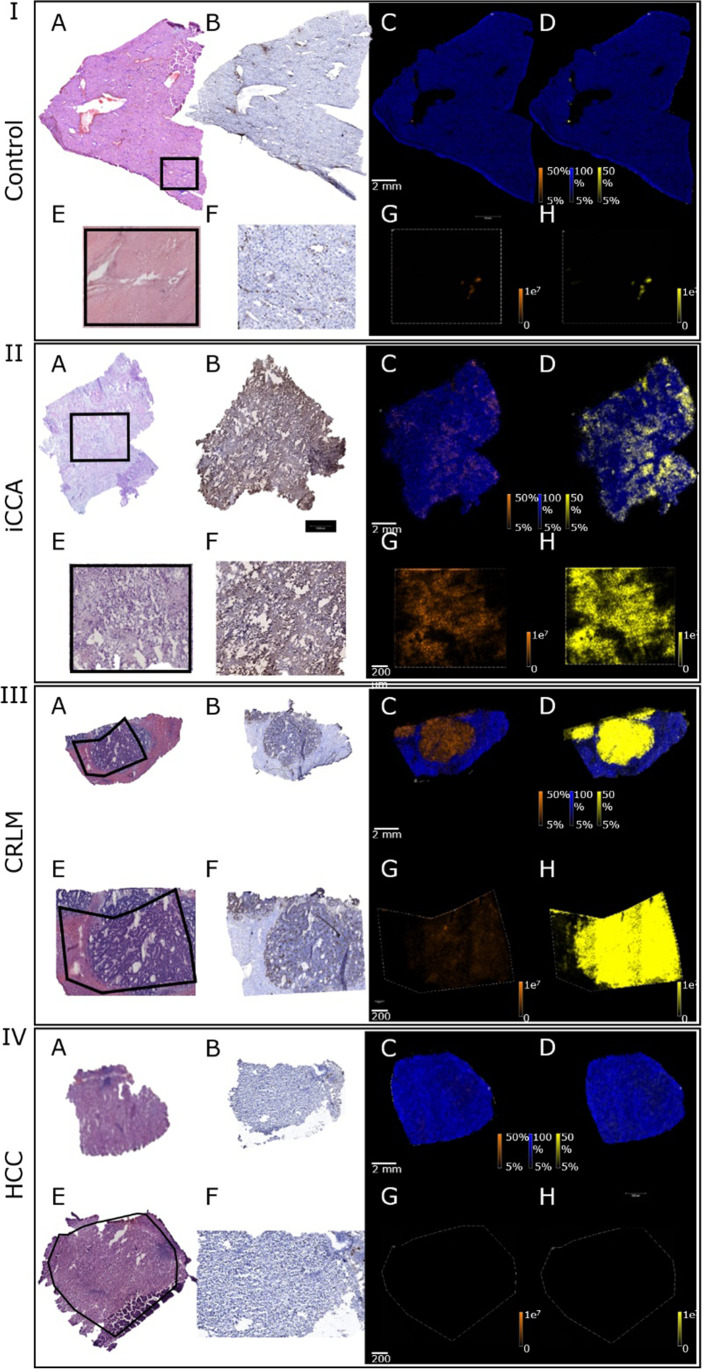
Sulfatides are present in tumor tissue of patients with intrahepatic cholangiocarcinoma. Mass spectrometry imaging was used to study the spatial distribution of sulfatides in control liver sections (**panel I**) and tumor tissue of patients with intrahepatic cholangiocarcinoma (**iCCA, panel II**), colorectal liver metastasis (**CRLM, panel III**) and hepatocellular carcinoma (**HCC, panel IV**). Imaged sections were stained with H&E (**panel A and E**), and adjacent sections were stained for the bile duct marker cytokeratin 19 (**panel B and F**). Molecular images were obtained by high-throughput screening of the entire section on a RapiFlex system (**panel C and D**), with a region of interest (black lined area in panel A and E) selected for further high resolution imaging on a Solarix FT-ICR (**panel G and H**). **[C]** Distribution of *m/z* 885.55 ± 0.1 (phosphoinositide [38:4], blue) as parenchymal marker, and *m/z* 876.62 ± 0.1 (ST [41:1], orange) as marker for bile ducts. **[D]** Distribution of *m/z* 885.55 ± 0.1 (phosphoinositide [38:4], blue) as parenchymal marker and *m/z* 892.63 ± 0.1 (ST-OH [41:1], yellow) as marker for the bile ducts. **[G]** Distribution of *m/z* 876.6240 ± 0.001 (ST [41:1], orange). **[H]** Distribution of *m/z* 892.6189 ± 0.001 (ST-OH [41:1], yellow). Representative images are shown

MSI was used to study the occurrence, and spatial distribution of sulfatides in control and liver tumor tissue. In liver tissue sampled distal from the tumor (*i.e.* CRLM or HCC), sulfatides were observed exclusively in the bile ducts (Fig. 1, panel I_GH_), in line with earlier findings [3]. This aspect led us to designate tumor-distal tissue here as control tissue. A total of 41 distinct sulfatides were identified in the bile ducts, with the hydroxylated species ST-OH [34:1] having the highest intensity (Supplemental Tables 2–4). In CRLM and iCCA, ST-OH [42:1] was most abundant, though distributed over the tumor area instead of being localized to the bile ducts. Over 40 additional sulfatide species were readily detected in tumor areas of iCCA and CRLM (Supplemental Tables 2–3), as depicted for ST-OH [41:1] in Fig. 1 (panel II_GH_ and III_GH_). This particular species was hardly observed in HCC tumor areas (Fig. 1, panel IV_GH_). In fact, only low intensities were observed for sulfatides in the HCC specimens. In general, hydroxylated sulfatides displayed a higher intensity than the non-hydroxylated equivalent (shown for ST-OH [41:1] in Fig. 1, panels I-IV_CDGH_). Note that a lipid marker for the hepatic parenchymal cells (*i.e.* phosphoinositide [38:4] [3]) was detected in all control and tumor specimens, without overlapping expression with tumor areas in iCCA and CRLM (Fig. 1, panels I-IV_CD_).

### Total sulfatide composition is largely similar in iCCA and control tissue

**Fig. 2:**
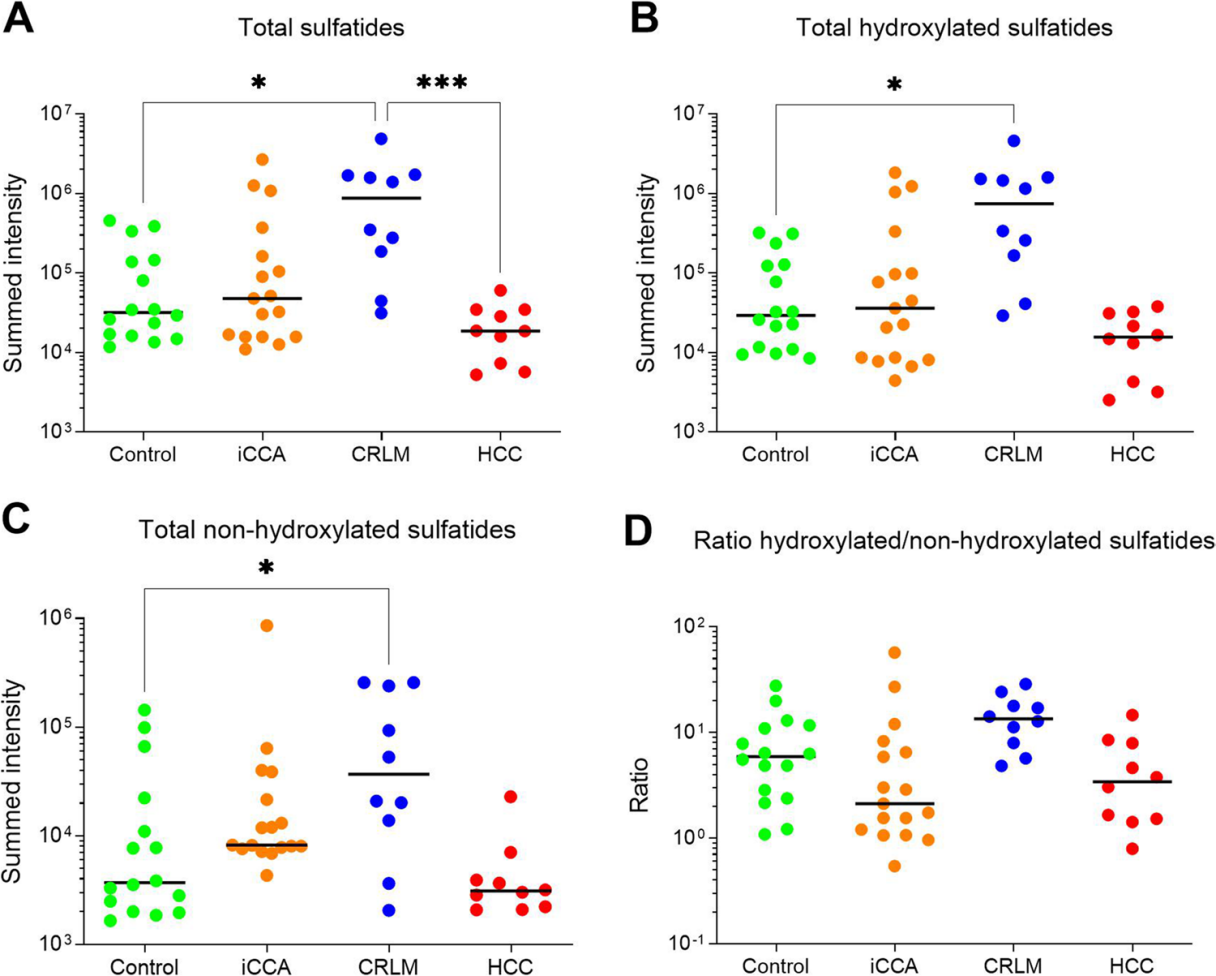
Total sulfatide abundance is elevated in tumor tissue of patients with colorectal liver metastasis. Summed sulfatide intensities per pixel from control regions (bile ducts, green) and tumor regions of intrahepatic cholangiocarcinoma (iCCA, orange), colorectal liver metastasis (CRLM, blue) and hepatocellular carcinoma (HCC, red) for all sulfatides (**panel A**), hydroxylated species (**panel B**) and non-hydroxylated variants (**panel C**), as well as the ratio between the latter two (**panel D**). Dots represent individual samples, with the median intensity depicted by a solid line. Note that all y-axis scales are logarithmic. Statistical significance for between-group comparisons is depicted. * denotes *P* < 0.05, *** denotes *P* < 0.001

Whereas the sulfatide-positive area was evidently larger in iCCA tissue (Fig. 1), the median summed sulfatide intensity per pixel was similar in comparison with bile ducts (47878 [15764–267010] vs. 31776 [16430–143506] in iCCA and controls, resp.; *P* = 1.00) (Fig. 2A). In contrast, median total sulfatide signal was notably higher (18–47 fold) in CRLM in comparison with control (*P* = 0.027) and HCC tissue (*P* < 0.001), with a trend to elevation relative to iCCA tissue (*P* = 0.068). Median total sulfatide intensity in HCC was comparable to control and iCCA. Note that there is substantial within-group variation in total sulfatide intensity, which is smallest in the HCC group.

In general, in each group hydroxylated sulfatide species appeared more abundant than non-hydroxylated variants. The median sum of both hydroxylated and non-hydroxylated sulfatides, as well as their ratio, was similar in iCCA relative to controls (Fig. 2BCD). The increased total sulfatide intensity in CRLM was due to elevations of both non-hydroxylated (median tenfold, *P* = 0.028) and hydroxylated (median 26 fold, *P* = 0.017) sulfatide species, with the latter having the largest impact in absolute terms. The ratio of hydroxylated over non-hydroxylated sulfatide species was not distinct in CRLM and control tissue. No compositional changes were apparent in HCC with regard to (non)-hydroxylated species when compared with controls.

**Fig. 3:**
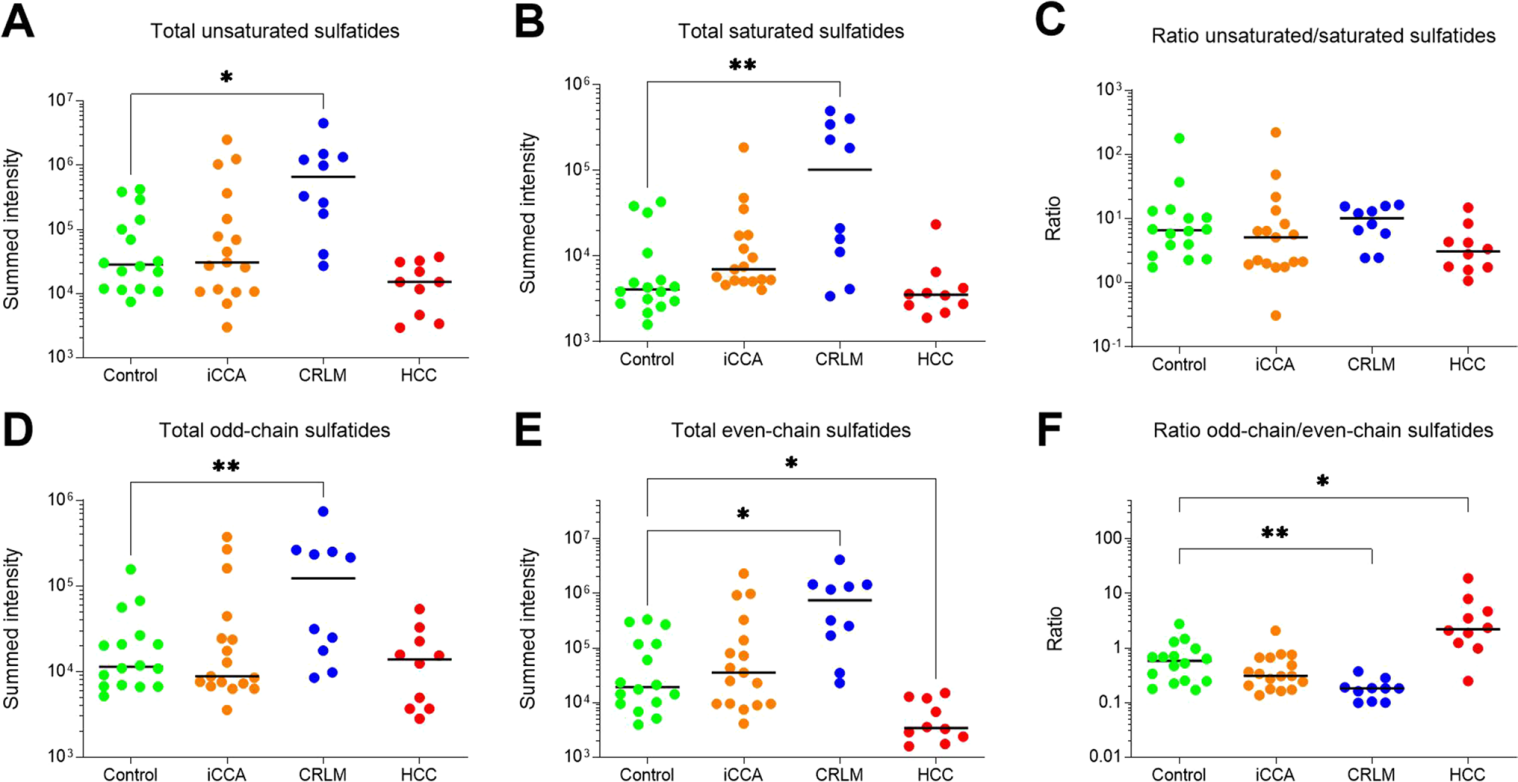
The ratio of unsaturated over saturated sulfatide species is indistinguishable in control tissue and tumor groups. Summed sulfatide intensities for unsaturated (**panel A**) and saturated species (**panel B**) and their ratio (**panel C**), as well as for odd-chain (**panel D**) and even-chain sulfatides (**panel E**) and their ratio (**panel F**) are depicted for the respective groups. Dots represent individual samples, with the median depicted by a solid line. Note that all y-axis scales are logarithmic. Statistical significance for between-group comparisons is depicted. * denotes *P* < 0.05, ** denotes *P* < 0.01

We observed identical patterns when looking at (un)saturated sulfatide species. In general, in each group unsaturated sulfatides were more abundant than saturated variants (Fig. 3). Median summed intensity of both unsaturated and saturated sulfatide species was similar in iCCA and controls (*P* = 1.00 and *P* = 0.067, resp., Fig. 3ABC). In CRLM, the elevated sulfatide levels were due to both rises in unsaturated (median 25 fold; *P* < 0.019) and saturated species (median 23 fold; *P* = 0.006). The ratio of unsaturated over saturated sulfatide species was indistinguishable in the three tumor groups and the control. There is considerable variation in this ratio, especially in the control and iCCA groups.

A final distinction was made between sulfatide species with even and odd acyl chain substitutions. In general, even-chain sulfatide appeared slightly more abundant in each group (Fig. 3DE). Increased levels of sulfatides in CRLM comprised both elevations of even-chain species (median 39 fold increase vs. control; *P* = 0.018) and odd-chain species (median 11 fold increase vs. control; *P* = 0.007). Relative to controls, the ratio of odd-chain to even-chain sulfatides was lower in CRLM (-3.2 fold, *P* = 0.009), while elevated in HCC (+ 3.8 fold, *P* = 0.043) (Fig. 3F).

We choose to not analyze between-group differences at the level of individual sulfatide species. It was noted though that sulfatides with C18 *N*-acyl chains (*e.g.* ST-OH [36:0]) were absent in all groups. This may reflect absent or low hepatic expression of ceramide synthases with C18 acyl length specificity (*i.e.* CerS1 and CerS4). Sulfatides with acyl chain lengths between C38-C44 comprised the dominant species in all groups (Supplemental Fig. 2), in line with the substrate specificity (C20-C26) of the major hepatic ceramide synthase CerS2 [21].

### Specimen subgroups have distinct sulfatide patterns

**Fig. 4:**
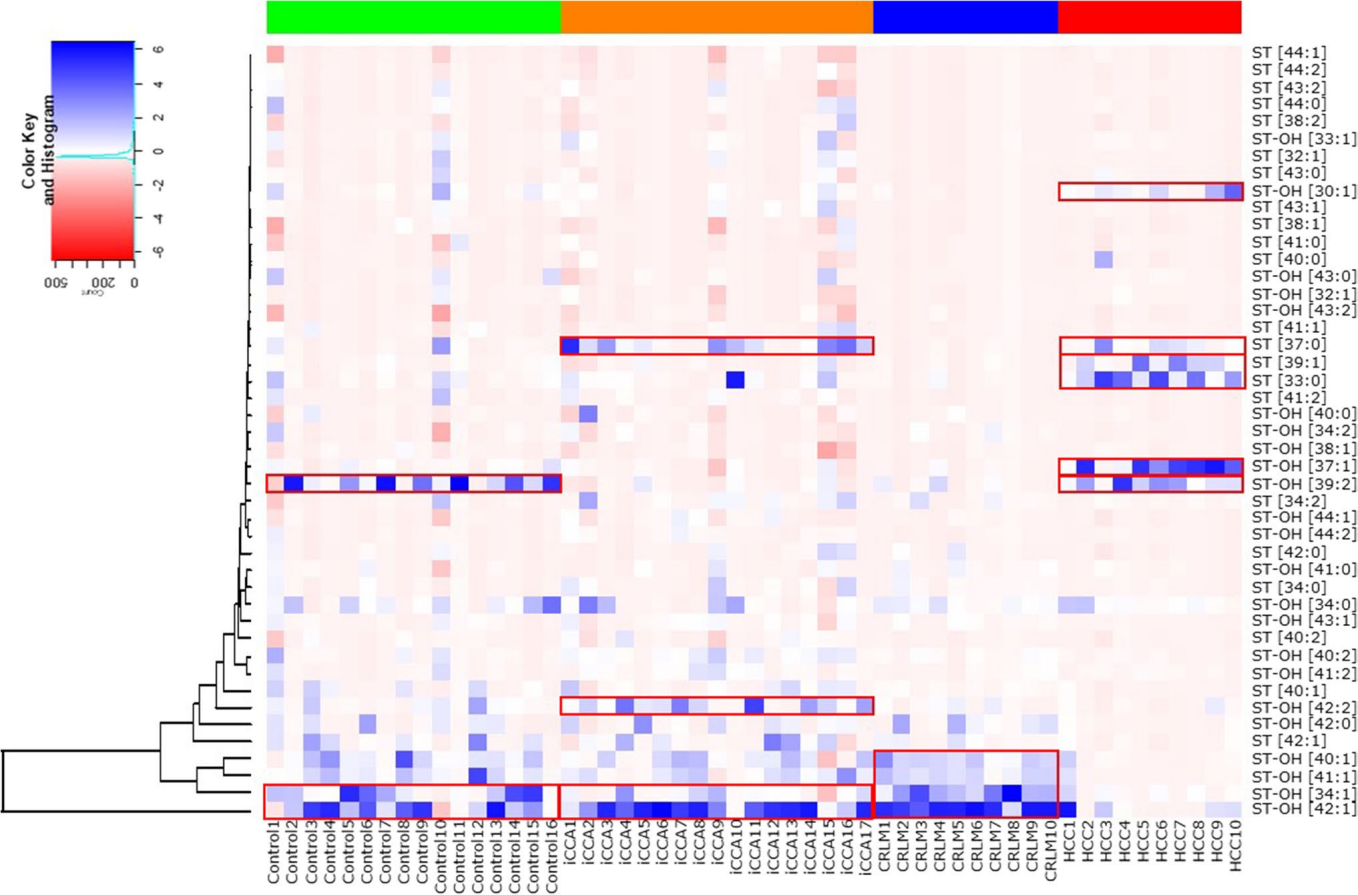
Specimen subgroups have distinct sulfatide patterns. Unsupervised hierarchical clustering of all analyzed samples was performed based on the intensities of constituent sulfatides. The clustergram depicts the scaled intensities of all detected sulfatides, sorted per group. Boxed regions (red coloring) are discussed in the text

Unsupervised hierarchical clustering showed an indicative grouping of the various specimens in distinct (sub)groups (Fig. 4). Overall sulfatide profiles differed between the various tumor types (see also Supplemental Table 4 and Supplemental Fig. 3). As particularly striking example, ST-OH [42:1] was highly abundant in tumor areas of CRLM or iCCA, but was hardly observed in HCC tumor areas (Fig. 4, Supplemental Table 4).

In case of the controls, a scattered image was obtained as some specimens show strong signals of ST-OH [39:2], whilst others displayed elevated levels of ST-OH [34:1] and ST-OH [42:1], which are dominant species in the CRLM and iCCA groups as well. The iCCA group showed inter-tumor heterogeneity, and the samples clustered in two (overlapping) groups. One subgroup showed elevated levels of ST [37:0] [10 out of 17], whilst the other group had elevated levels of ST-OH [42:2] [10 out of 17]. ST [37:0] was also observed being upregulated in a portion of HCC tumors. The CRLM tumors showed a remarkable homogeneity amongst the 10 examined specimens. Nearly all samples contained upregulated levels of four hydroxylated sulfatide species, viz*.* ST-OH [34:1], ST-OH [40:1], ST-OH [41:1] and ST-OH [42:1]. The abundance of these compounds in iCCA and controls was much more heterogenous.

A number of sulfatides were clearly upregulated in the HCC specimens, viz*.* ST [33:0] (6 out of 10) and ST [39:1] (5 out of 10), as well as ST-OH [37:1] (7 out of 10) and ST-OH [30:1] (4 out of 10). ST [37:0] was clearly enhanced in some HCC samples, though also upregulated in part of the iCCA tumors. ST-OH [39:2] was observed in both HCC tumors and the majority of control samples.

To evaluate intra-tumor heterogeneity, we analyzed iCCA specimens from 6 patients where a second specimen of (a different part of) the tumor was available. Intensities of (non)hydroxylated sulfatide species in the tumor area were similar (Supplemental Fig. 4). Likewise, spatial distribution of sulfatide species was grossly comparable.

### The ratio of unsaturated to saturated sulfatides is related to shorter disease-free survival in iCCA

We conducted correlation analysis to evaluate whether the sulfatide pattern is linked to tumor biology and/or clinical outcomes in patients with iCCA. To this end, (disease-free) survival, tumor recurrence, tumor grade and tumor size/extension and lymph node involvement (T and N components in TNM staging system) were correlated to the total intensities of sulfatide classes and ratios derived thereof.

The tumor differentiation state (G grade), tumor stage and lymph node status (TNM classification) were not related to total sulfatide intensity or compositional features (Supplemental Figs. 5–7).

**Fig. 5:**
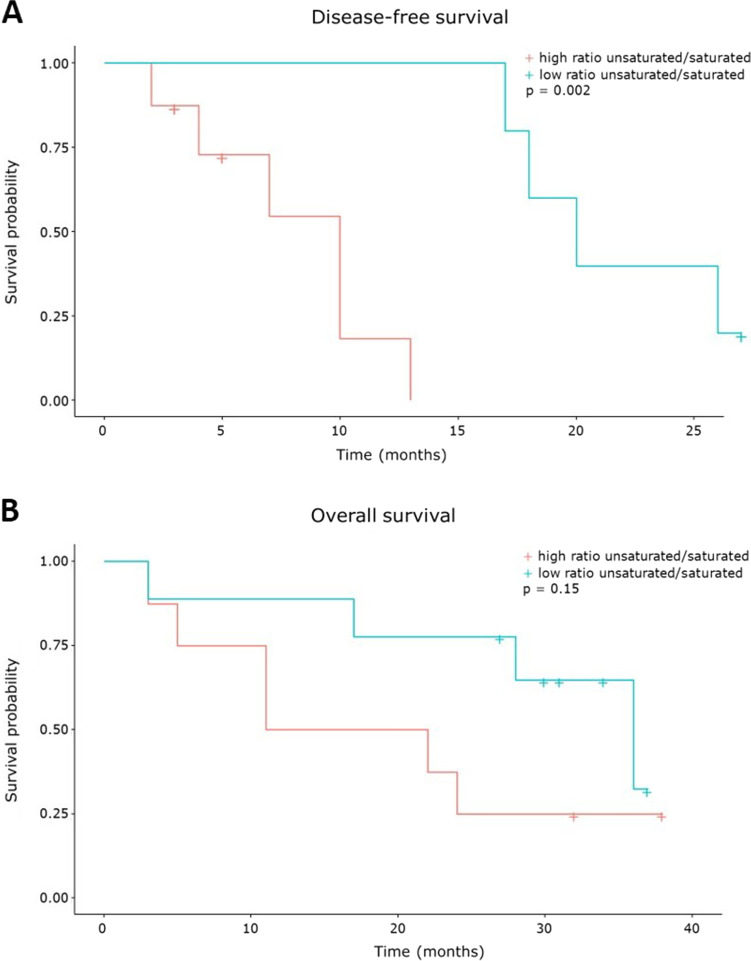
A high ratio of unsaturated over saturated sulfatides in iCCA tumor area is associated with earlier tumor recurrence. Disease-free survival (*P* = 0.002) (**A**) and overall survival (*P* = 0.15) (**B**) in relation to the saturation ratio (unsaturated over saturated sulfatides) in patients with iCCA. Red is high ratio, blue is low ratio, groups separated on 50% quantile

Tumor recurrence within the follow-up period of 2 years, was noted in 10 out of 15 patients who underwent actual liver resection. All operated patients received adjuvant chemotherapy (capecitabine or gemcitabine plus cisplatin). Note that two patients received an exploratory laparotomy but were not resectable at first surgery (Supplemental Table 1). A significant correlation was found between disease-free survival and saturation index (*P* = 0.002) (Fig. 5A). Patients with a high ratio of unsaturated to saturated sulfatides showed earlier tumor recurrence, with a median time to recurrence of 10 and 20 months in high and low ratio groups, respectively. Disease-free survival was not linked with other sulfatide compositional features (Supplemental Table 5).

There was no in-hospital mortality or mortality within 90 days, in iCCA patients undergoing actual liver resection. Cancer-related death within 2 years of operation occurred in 8 out of 17 patients, with demise of two other patients unrelated to their malignancy. The saturation ratio was associated with overall survival in Cox proportional hazard analysis (*P* = 0.041, Supplemental Table 5), but not in Kaplan–Meier analysis (Fig. 5B). No other correlations were apparent for sulfatide characteristics and overall or disease-specific survival (Supplemental Table 5).

## Discussion

We aimed to determine if sulfatides are present in tumors originating from the intrahepatic bile duct, and whether sulfatide compositional features are related with clinical outcomes. Here, we demonstrate for the first time that sulfatides are present in tumor tissue of patients with iCCA. Although the sulfatide-positive area was evidently larger in iCCA tissue, summed sulfatide intensity per pixel was similar in comparison with bile ducts. Likewise, global composition of sulfatides in iCCA was not distinct from control tissue. Strikingly though, a high ratio of unsaturated to saturated sulfatides was associated with reduced disease-free survival in patients with iCCA.

In line with our earlier observation [3], sulfatides were specifically present in the bile ducts but were not detected in the parenchyma in tumor-distal control tissue (Fig. 1). In iCCA the entire tumor area was sulfatide-positive, with a total of 44 distinct sulfatide species detected in widely varying intensities (Fig. 4, Supplemental Tables 2–4). Considerable inter-tumor variability in total sulfatide intensity was observed in iCCA (Fig. 2A). Results from a second tumor region (available for 6 patients) showed no distinct changes in sulfatide intensities between the two replicated regions (Supplemental Fig. 4), demonstrating the robustness of the method and indicating that inter-tumor variation was not due to sampling location. Despite reported links with aggressiveness of tumors, no correlations between total sulfatide intensity with overall and disease-free survival (Supplemental Table 5) or tumor biology (*i.e.* T and N component in TNM stage, Supplemental Figs. 5–7) were apparent in iCCA. When looking at distinct classes of sulfatide species, relative abundance of unsaturated over saturated species was found to be associated with shorter disease-free survival (Fig. 5A). The molecular events linking sulfatide saturation index with earlier iCCA tumor recurrence are unclear yet.

It is tempting to speculate that in iCCA tumors with an elevated ratio of unsaturated over saturated sulfatides, enzymes coding for desaturases (*e.g.* the Δ9-stearoyl desaturase 1, SCD1) are elevated, thus, promoting incorporation of unsaturated acyl chains in ceramide and eventually showing up in sulfatides. Increased expression of the lipogenic enzyme SCD1 has been observed in cancer cells originating from a variety of tissues, and this has been linked with enhanced tumorigenic properties [22, 23]. Inhibition of SCD1 was shown to inhibit cell proliferation in among others lung cancer cell lines and in cell lines and xenografts of lipogenic-subtype pancreatic tumors [22, 24]. SCD1 inhibition is considered as therapeutic target in the treatment of cancer, and preclinical studies are ongoing. Inter-tumor variation in expression of the dihydroceramide Δ4-desaturases DEGS1 and DEGS2 that introduce a double bond in the sphingolipid backbone to generate ceramide, may also contribute to heterogeneity in the sulfatide saturation index in the iCCA group. Detection of saturated sulfatides (*e.g.* ST [34:0], with d18:0 as tentative sphingoid backbone and a C16:0 N-acyl chain) suggests that dihydroceramides can be further metabolized to more complex sphingolipids. DEGS inhibition results in diminished proliferation of various cell types *in vitro* [25]. It is thus conceivable that the balance between unsaturated and saturated sulfatides impacts on cell growth. It remains to be determined whether SCD1/DEGS inhibition is of benefit to patients with iCCA, which are typically diagnosed in an advanced stage of the disease.

The presence of sulfatides in liver tumor tissue was not restricted to iCCA, but also observed in tumors originating from liver parenchymal cells (HCC) or the colon (CRLM). In the latter group, total sulfatide intensities were notably higher (up to 47-fold) relative to control and HCC tumor tissue. All structural classes (*i.e.* sulfatides containing (non)hydroxylated, (un)saturated or even/odd chain *N*-acyl groups) contributed to elevated sulfatide levels in CRLM. Elevated expression of sulfatides was previously reported for primary colon carcinoma and cell lines derived thereof, and this was associated with increased metastatic potential [26, 27]. Apparently, sulfatide overexpression was maintained upon metastatic spread to the liver. During the follow-up period, tumor recurrence occurred in 6 out of 10 patients with CRLM, with an all cause-mortality of 50% in this group. Cox proportional hazard analysis revealed no significant associations between sulfatide compositional features and overall or disease-free survival in the CRLM group (Supplemental Table 6).

It is conceivable that enhanced expression of biosynthetic enzymes (*e.g.* GAL3ST1) contributes to elevated sulfatide levels in CRLM. It should be noted though that no direct comparison could be made with normal colon and primary colon cancer tissue, and the different tissue origin may account for the elevated levels in comparison with control and primary liver tumor tissue (*i.e.* HCC). Previously, overexpression of *GAL3ST1* was demonstrated in the context of renal clear cell carcinoma and ovarian cancer [12, 28, 29]. Given that both hydroxylated and unsaturated sulfatide species had substantial quantitative contributions to elevated sulfatide content in CRLM, overexpression of the fatty acid 2-hydroxylase FA2H and fatty acid/sphingolipid desaturases is likely in CRLM.

Although we did not observe correlations between sulfatide hydroxylation parameters and survival in CRLM patients, elevated levels of hydroxylated sulfatides may impact on tumor biology [30–33]. Empirical evidence for such action is thus far limited and circumstantial, and the literature indicates that effects may be tumor dependent. Hydroxylation of glycosphingolipids was associated with a highly tumorigenic and invasive phenotype of urothelial cell lines [34]. Moreover, elevated levels of hydroxylated glycosphingolipids were associated with chemoresistance of ovarian carcinoma cell lines. On the other hand, FA2H inhibition resulted in reduced growth and enhanced cisplatin sensitivity of gastric cancer cells [35]. Likewise, a tumor suppressive role has been proposed for FA2H in breast cancer [30]. Note that in these latter instances, changes were not directly linked to hydroxylated glycosphingolipids, and could have been effects of hydroxylated fatty acid in free form or after incorporation in lipids other than glycosphingolipids. Likewise, in the context of colorectal cancer, fatty acid 2-hydroxylation was reported to inhibit tumorigenic properties of colorectal cancer cells [36]. Therapeutic potential of FA2H and/or SCD1/DEGS modulation in CRLM remains to be determined.

The third liver tumor in which we observed sulfatides was HCC. Interestingly, the sulfatide species predominating in control bile ducts and iCCA and CRLM tumor tissue, viz. ST-OH [42:1], was virtually absent in HCC tissue (Fig. 4). Instead, the odd-chain species ST-OH [37:1] was most abundant in HCC (Fig. 4, Supplemental Table 4). Of note, odd-chain sulfatides could be detected in all groups, but their relative abundance (Fig. 3) was increased in HCC. This is reflected in the clustergram (Fig. 4), where HCC appears to have an odd-chain sulfatide signature. Larger studies are required to test specificity and clinical relevance of this putative signature, *e.g.* in differential diagnosis of HCC and iCCA which can be problematic upon clinical imaging or in histopathological diagnosis [37, 38]. It is tempting to speculate that peroxisomal alpha-oxidation, which is prominent in peroxisome-dense hepatocytes, results in generation of odd-chain fatty acids that can be incorporated into the cellular lipid pools, including ceramides and sulfatides. During follow-up, tumors recurred in 4 out of 10 patients with HCC, and all cause-mortality in this group was 20%. Cox proportional hazard analysis revealed no significant associations between sulfatide compositional features and overall or disease-free survival in these patients (Supplemental Table 7). In an earlier study high expression of sulfatides (based on immunoreactivity) in HCC tumor tissue and HCC-derived cell lines was linked with metastatic behavior [39]. Furthermore, enhanced synthesis of sulfatides through overexpression of the sulfotransferase GAL3ST1 increased metastatic potential of HCC cell lines [39]. Further exploration of a tumorigenic link with aspects of sulfatide metabolism is also warranted for HCC.

To our knowledge, this is the first report on spatial sulfatide distribution in hepatobiliary tumors. The expression of sulfatides in the three studied tumor types raises questions on their involvement in tumorigenesis and tumor progression. Sulfatides have been recognized as adhesive molecules in the outer leaflet of the plasma membrane, that bind to specific target proteins [4, 12]. For instance, sulfatides serve as native ligands for P-selectin on the cell surface of platelets, thus, contributing to metastasis of breast cancer cells [5, 40]. Sulfatides are furthermore components of lipid rafts, which are dynamic signaling hubs that control cell proliferation and differentiation [41, 42]. It is conceivable that hydroxylation and unsaturation(s) in the *N*-acyl chain of sulfatides, affects its packing in lipid rafts and/or lipid raft dynamics/function. In the context of clear cell renal carcinoma, enhanced sulfatide-mediated platelet binding protected tumor cells from cytotoxic assaults by Natural Killer cells. Accordingly, sulfatides may contribute to evasion of a tumor-directed immune response. Increased expression of GAL3ST1 was associated with decreased survival in primary clear cell renal carcinoma.

Whereas ceramides are generally considered to induce apoptosis, its galactosylated derivative βGalCer has anti-apoptotic effects in breast cancer cells [12, 40]. Likewise, the lactosylated derivatives (i.e. LacCer) appear to act as pro-survival signal in the context of fluke-associated CCA, where accumulation of hydroxylated LacCer[d18:1-h23:0] was associated with a drastic reduction in patient survival [16]. With regard to sulfatides, available evidence indicates that anti- or pro-apoptotic actions of sulfatides are context specific. It has been reported that sulfatides act as pro-apoptotic molecules, making cancer cells more prone to environmental stressors such as hypoxia and anticancer drugs [43, 44]. Sulfatides within tumors may promote apoptotic cell removal and alter the phenotype of tumor-associated macrophages [45, 46]. On the other hand, enhanced synthesis of sulfatides through overexpression of GAL3ST1 results in increased metastatic potential of tumor cells, indicating diversion of apoptotic to pro-survival signaling [5]. Further research is required to address the role of sulfatides in hepatobiliary tumors, and a potential therapeutic role of modulating sulfatide metabolism.

Our study has several limitations. Firstly, the iCCA samples were obtained from patients operated 2–3 years ago, and follow-up after discharge from hospital was limited to 2 years. Information on longer-term survival is not available for these and the CRLM/HCC patients. Secondly, the tumor samples were in 15 out of 17 cases obtained from resectable patients, and this could result in bias to a more favorable outcome and/or tumor biology. Only patients with non-locally advanced tumors (typically less aggressive tumors with less metastatic potential) are considered for surgical treatment. Overall survival increases from less than one year for inoperable patients, to a median survival of 27 months for patients with iCCA undergoing surgery (5-year survival between 20 and 40%). Thirdly, the sample size was relatively small and unbalanced. This is especially reflected in the tumor grades, with only a single patient having a grade 1 tumor and higher-grade tumors being limited to five patients each, and three tumors classified as grade 2–3 (Supplemental Table 1). With the achieved sample sizes it is realistic that the study is underpowered to detect differences in survival outcomes in relation to sulfatide compositional features. The statistical findings should therefore be regarded as indicators for further investigations, since at least an effect on disease-free survival was observed in patients with iCCA. Validation of the initial findings in independent patient cohorts of adequate size, will be necessary. Given the distinct presence of sulfatides in the tumor but not the tumor-distal area, a potential implication of our findings is the (real-time) monitoring of sulfatides during resection, to assess tumor-free margins during surgery in patients with hepatobiliary tumors as is currently being validated for other tumor types such as *e.g.* breast cancer and thyroid cancer [47–49].

In conclusion, expression of sulfatides was observed in tumor tissue of patients with iCCA, HCC and CRLM. In patients with iCCA, a high ratio of unsaturated to saturated sulfatide species was associated with reduced disease-free survival. In CRLM, markedly elevated levels of sulfatides were apparent, along with increased fractions of hydroxylated and unsaturated sulfatide species. HCC was marked by absence of sulfatide species common to bile ducts and iCCA and CRLM tissue, and relative abundance of odd-chain sulfatides. Follow-up studies are required to confirm initial links between sulfatides and survival in iCCA .

## Supplementary Information

Below is the link to the electronic supplementary material.

Supplementary file1 (DOCX 1.57 MB)

## Data Availability

The analyzed data sets generated during the study are available from the corresponding authors upon reasonable request.

## Abbreviations

AUC: Area under the curve
CCA: Cholangiocarcinoma
CRLM: Colorectal liver metastases
DEGS: Delta 4-desaturase sphingolipid
FA2H: Fatty acid 2-hydroxylase
FT-ICR: Fourier-Transform ion cyclotron resonance
GalCer: Galactosylceramide
HCC: Hepatocellular carcinoma
iCCA: Intrahepatic CCA
LacCer: Lactosylceramide
m/z: Mass over charge ratio
MALDI: Matrix-assisted laser desorption ionization
MS: Mass spectrometry
MSI: Mass spectrometry imaging
SCD1: Stearoyl desaturase 1
ST: Sulfatide
ST-OH: Hydroxylated sulfatide
