# Supplementary material for: Tumor ratio of unsaturated to saturated sulfatide species is associated with disease-free survival in intrahepatic cholangiocarcinoma
Source: Cell Oncol (Dordr). 2023 Jan 11;46(3):629–42. doi: 10.1007/s13402-022-00766-6 (PMC10205872; doi:10.1007/s13402-022-00766-6)
Supplement: Supplementary file 1 — Supplementary file1 (DOCX 1.57 MB) [file 13402_2022_766_MOESM1_ESM.docx]

**Supplemental Information to:**

**Tumor ratio of unsaturated to saturated sulfatide species is associated with disease-free survival in intrahepatic cholangiocarcinoma.**

Lennart Huizing^1,*^, Lin Chen^2*^, Anjali A. Roeth^2,3^, Lara R. Heij^3^, Bryn Flinders^1^, Stefan A.W. Bouwense^2^ Benjamin Balluff^1^, Ulf P. Neumann^2,3^, Ron M.A. Heeren^1^, Steven W.M. Olde Damink^2,3^, Rob J. Vreeken^1,4,#^, Frank G. Schaap^2,3,#^.

^*^Equally contributing first authors

^#^Shared senior authors

^1^Maastricht Multimodal Molecular Imaging Institute (M4I), Maastricht University, Universiteitssingel 50, 6229 ER Maastricht, The Netherlands.

^2^Department of Surgery, Maastricht University Medical Center and NUTRIM School of Nutrition and Translational Research in Metabolism, Maastricht University, Maastricht, The Netherlands.

^3^Department of General, Visceral and Transplantation Surgery, RWTH University Hospital Aachen, Aachen, Germany.

^4^Janssen Research & Development, Turnhoutseweg 30, 2340 Beerse, Belgium.

**Corresponding author:**

Frank G. Schaap, PhD

Maastricht University

Department of Surgery

NUTRIM School of Nutrition and Translational Research in Metabolism

PO BOX 616

6200 MD Maastricht

The Netherlands

Phone: +31 43 388 4502

Fax: +31 43 388 4154

Email: frank.schaap@maastrichtuniversity.nl


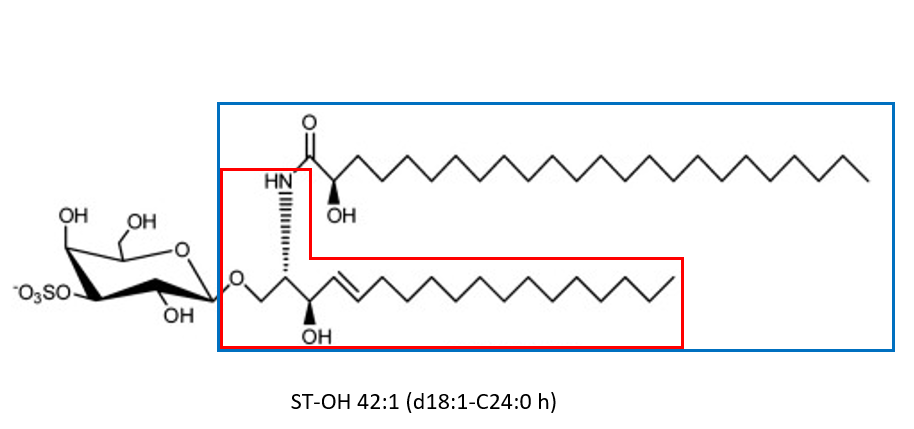


**Supplemental Figure 1.** General structure of a sulfatide with the prevailing sphingoid base (d18:1) in humans (depicted in red rectangle). d18:1 denotes a dihydroxy sphingoid base with a total of 18 carbon atoms and one double bond. *N*-acylation of the sphingoid base results in formation of a ceramide (blue rectangle). In sulfatides, the hydroxy groups at C1 of the sphingoid base is in β-linkage with a galactose residue (βGalCer) and has a 3’ sulphate group. Depicted here is ST-OH 42:1, with a d18:1 sphingoid backbone and a saturated *N*-acyl chain with 24 carbon atoms that is hydroxylated at C2 (C24:0 h).


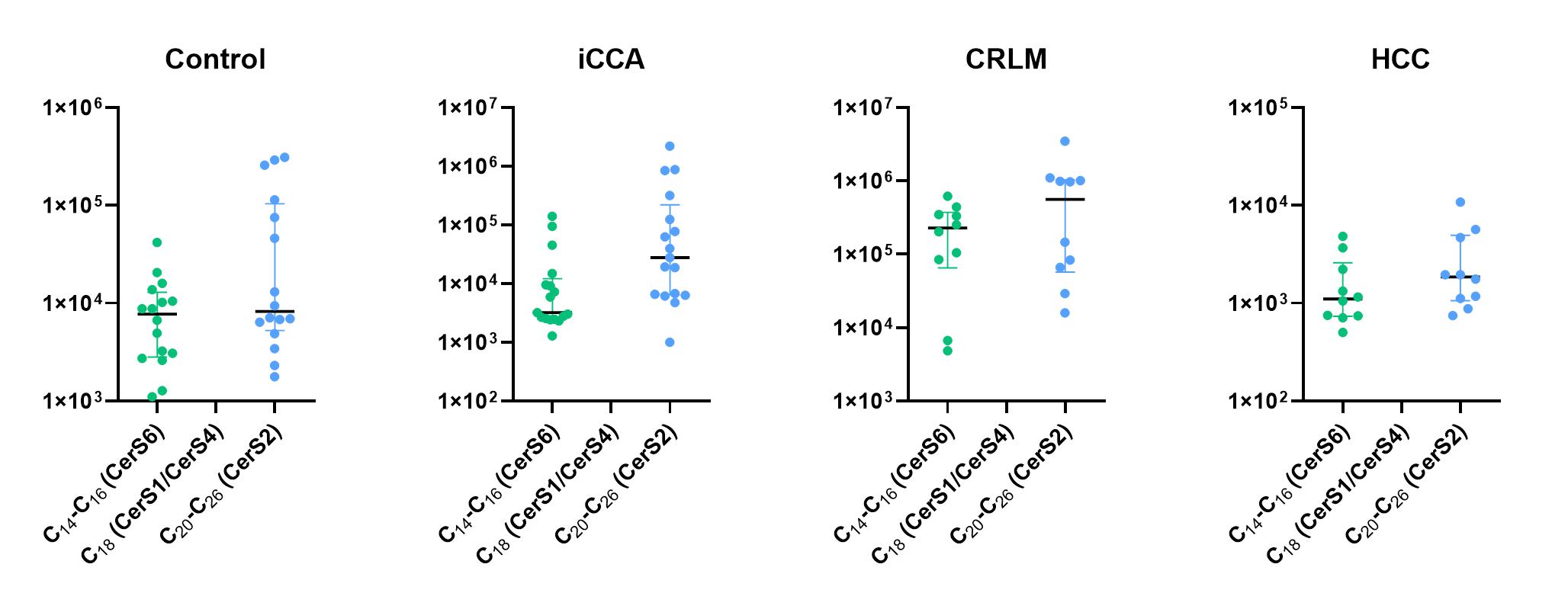


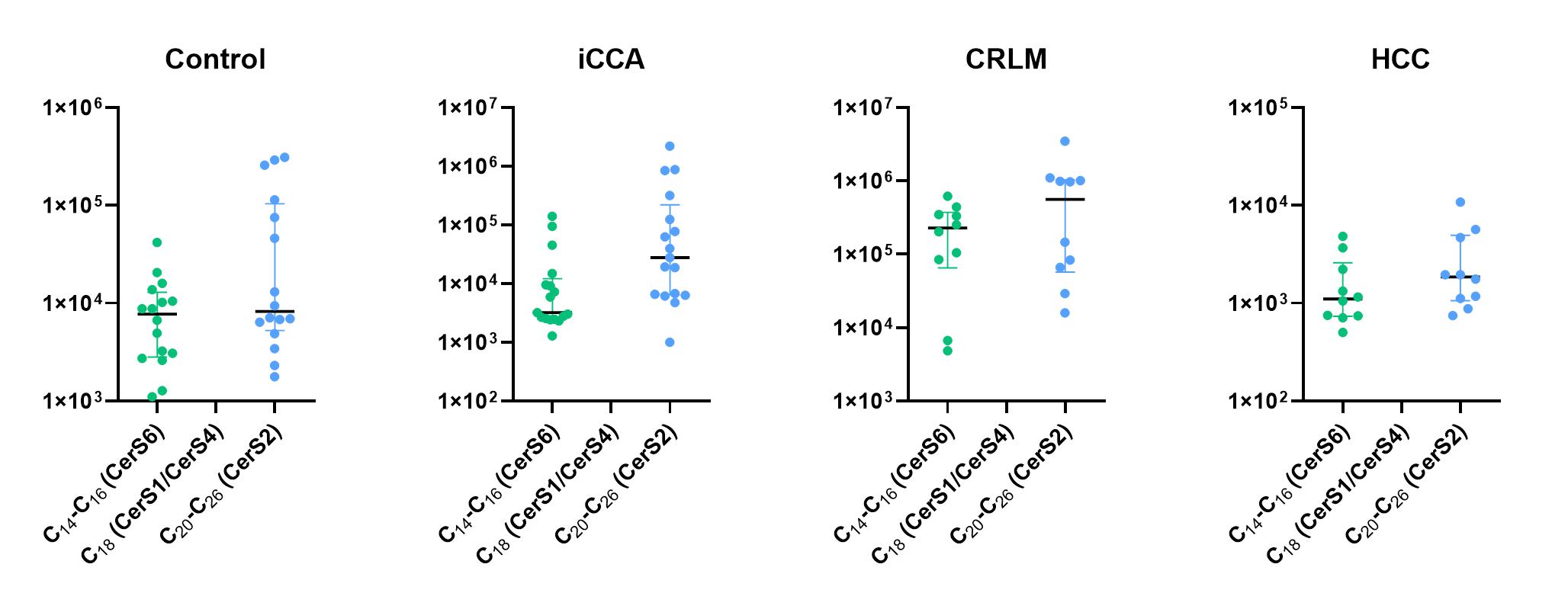


**Supplemental Figure 2**. Sulfatides with *N*-acyl chain lengths between C_20_-C_26_ comprise the dominant species in all groups. *N*-acyl chain length preferences of the indicated ceramide synthases (CerS) is according to Levy *et al.* [21].


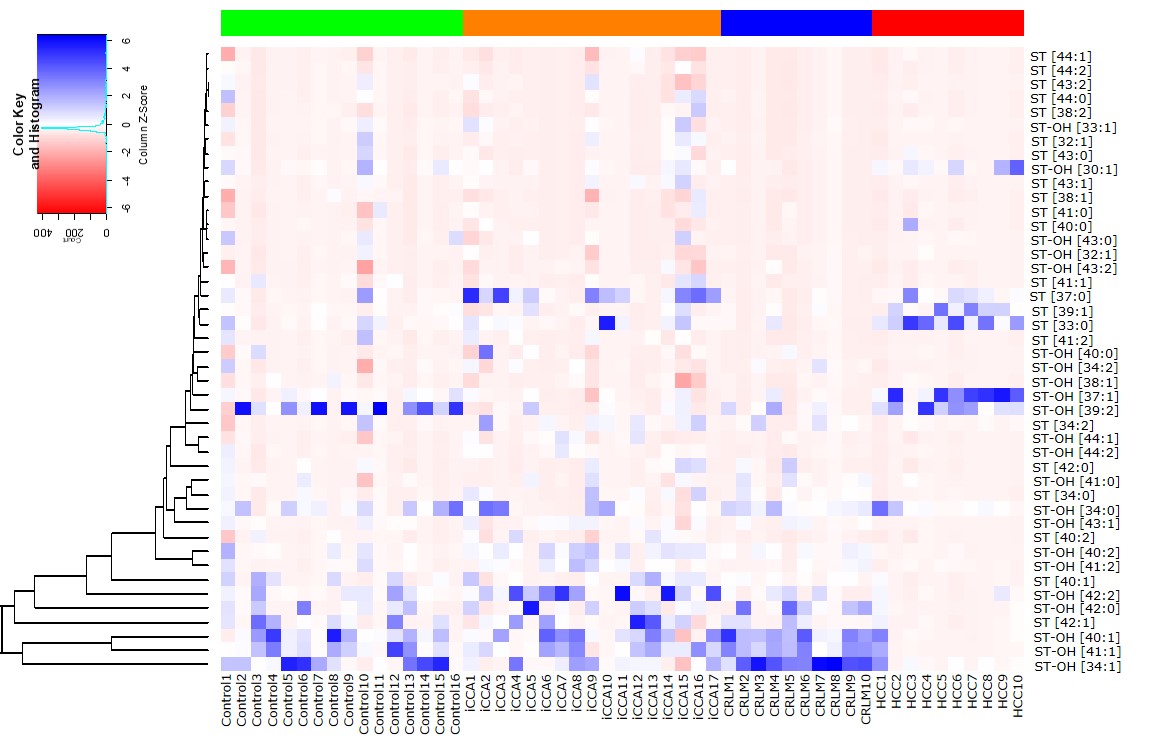


**Supplemental Figure 3.** Clustergram of the scaled intensities of all detected sulfatides but ST-OH [42:1], sorted per group.


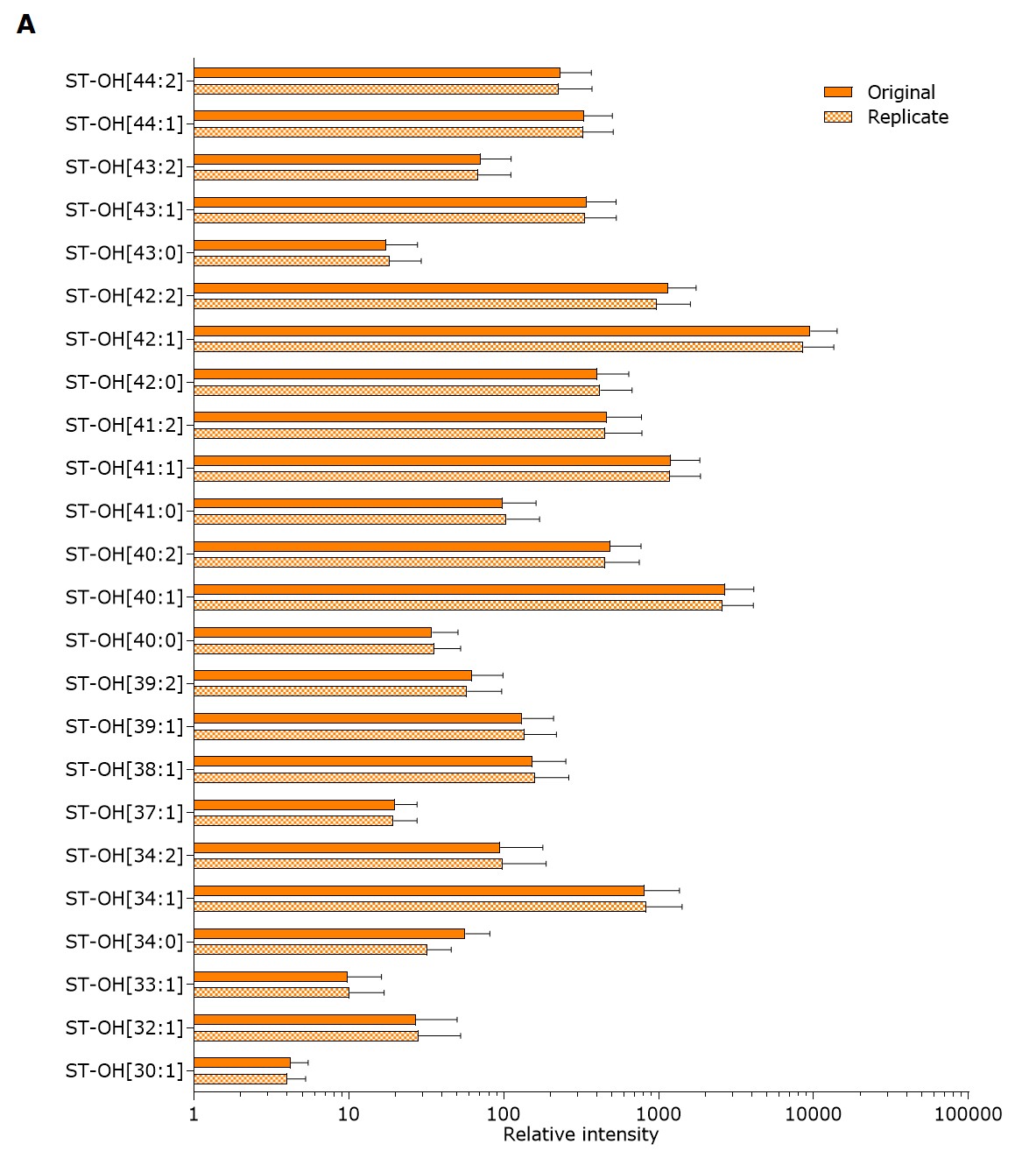

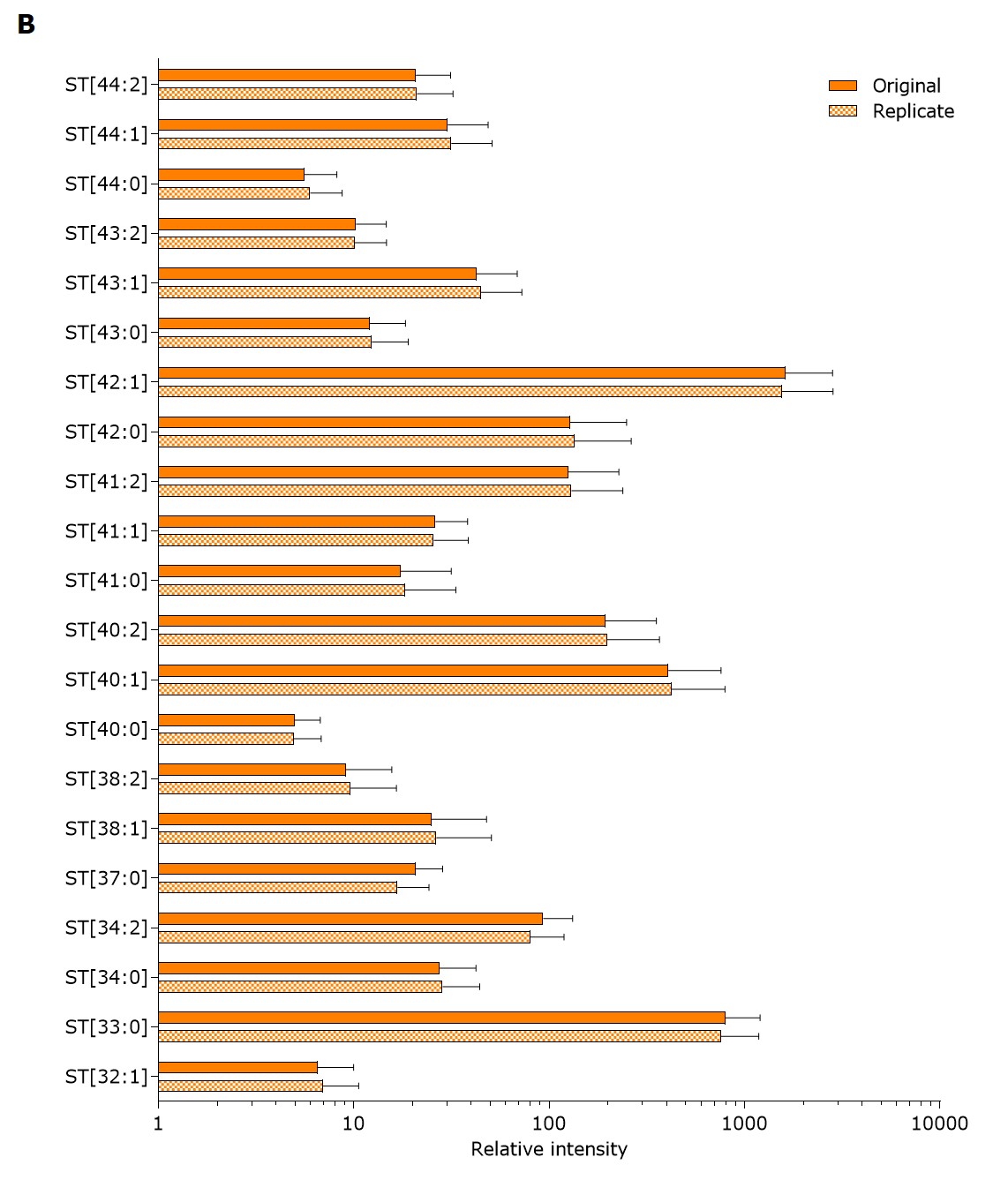


**Supplemental Figure 4.** Comparison of relative sulfatide intensity between original and six replicated iCCA samples for hydroxylated (**A**) and non-hydroxylated (**B**) sulfatides. Bars depict mean + SEM. Note that the x-axis is scaled logarithmically.

**
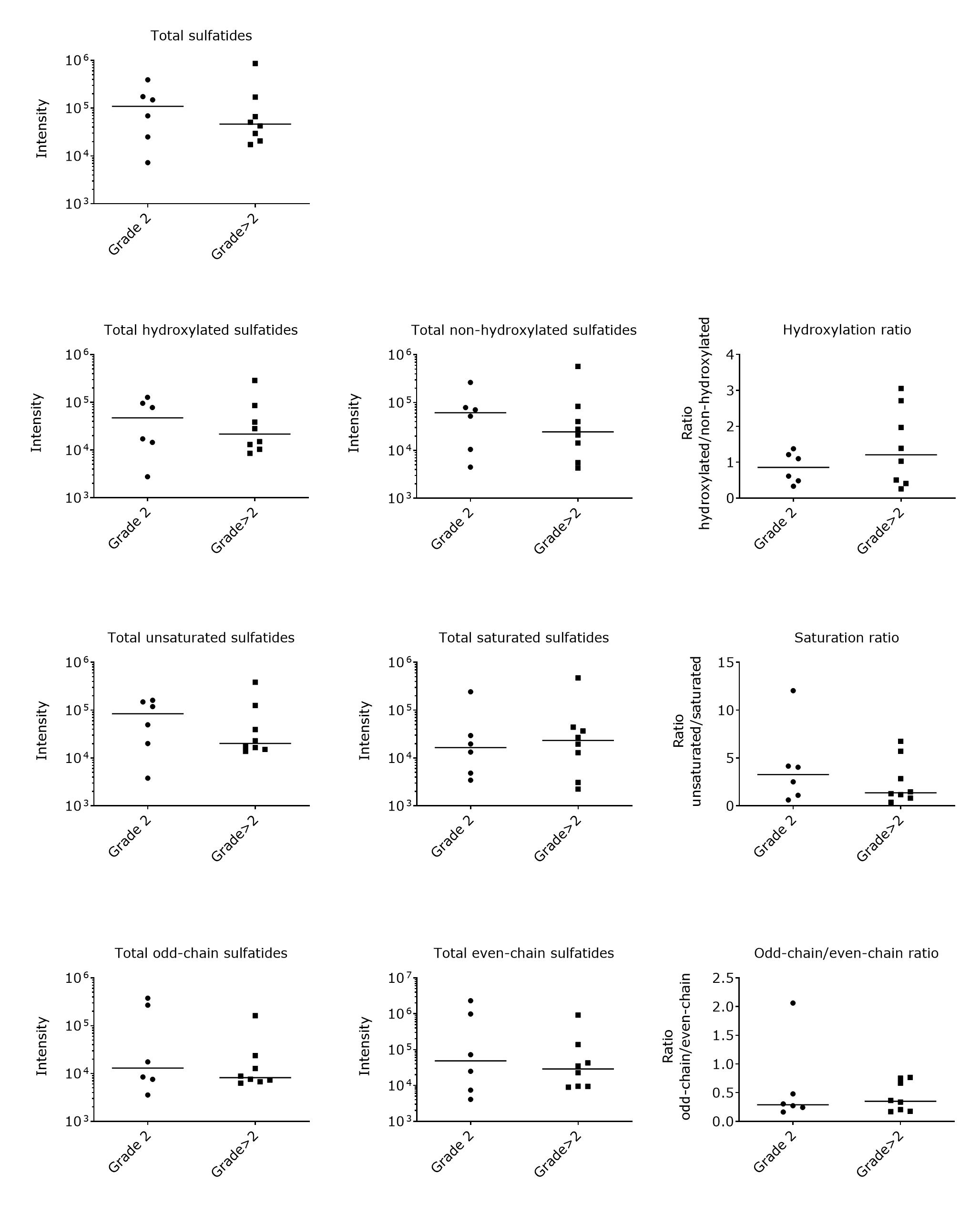
**

**Supplemental Figure 5**. Correlations between iCCA tumor grade and depicted sulfatide features. TNM classification and tumor grade was available for 15 patients with iCCA. Note that a single tumor specimen had grade 1, and was not included in the analysis. No correlations between ST features and tumor grade were observed.

**
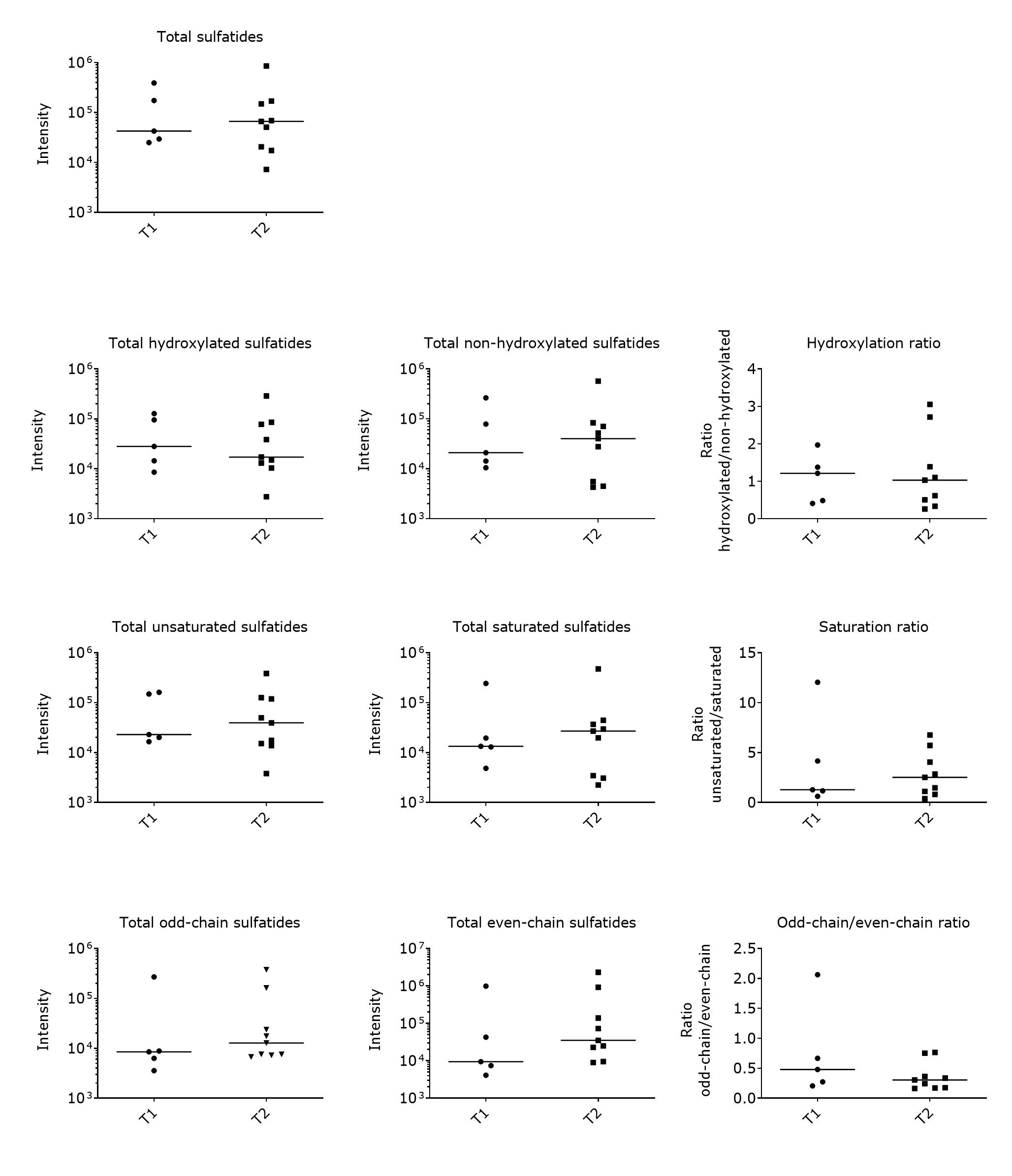
**

**Supplemental Figure 6**. Correlations between iCCA tumor stage and depicted sulfatide features. TNM classification and tumor grade was available for 15 patients with iCCA. Note that a single tumor specimen had stage 3, and was not included in the analysis. No correlations between ST features and tumor size and/or extent were observed.


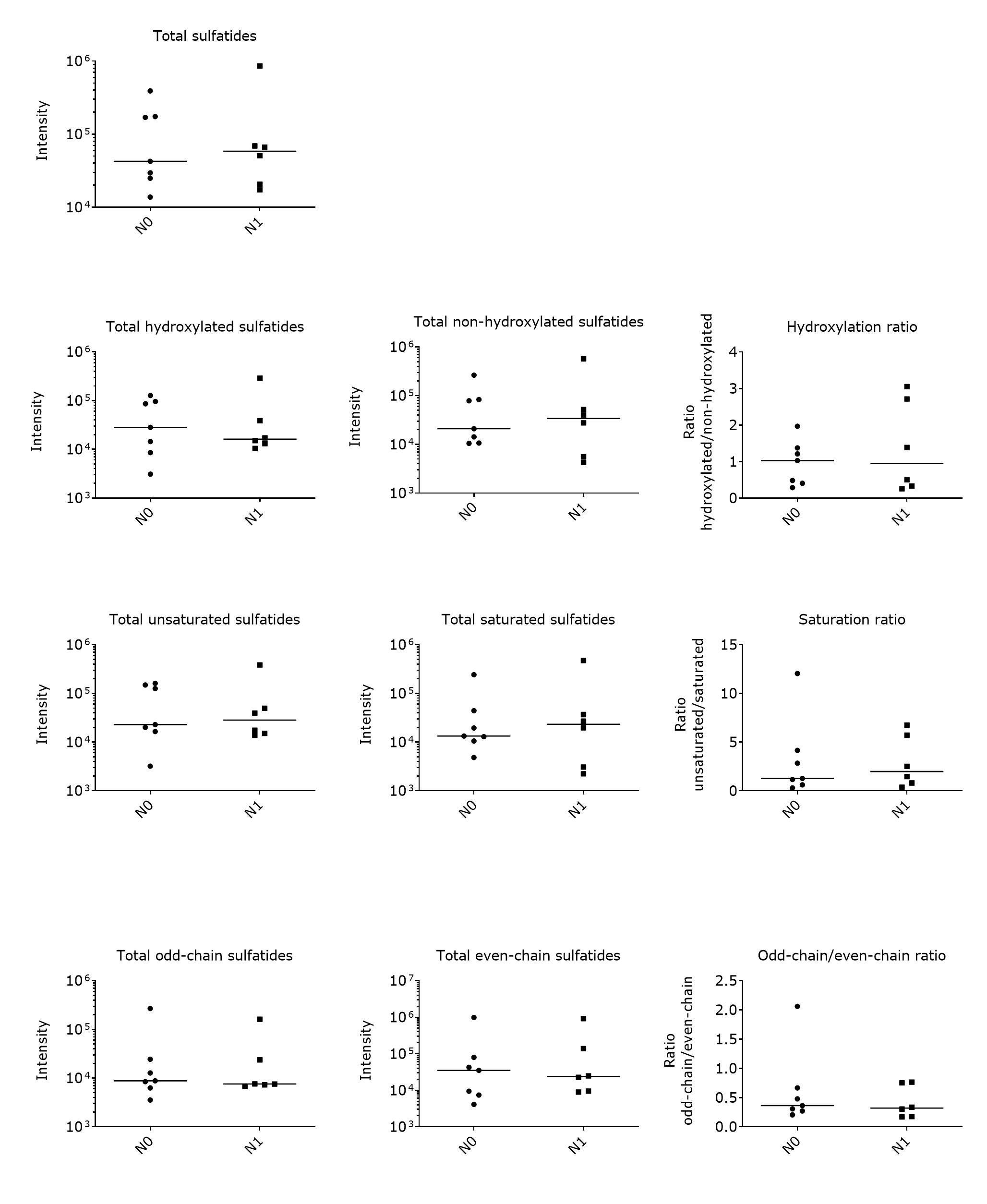
**Supplemental Figure 7**. Correlations between lymph node status and depicted sulfatide features. TNM classification and lymph node status was available for 13 patients with iCCA. No correlations between ST features and lymph node status were observed.

| **Supplemental Table 1. Characteristics of patients with intrahepatic CCA.** | | | | | | | | |
| --- | --- | --- | --- | --- | --- | --- | --- | --- |
|  | **Age (years)** | **T** | **N** | **G** | **Tumor recurrence** | **Mortality** | **Overall survival (months)** | **Disease-free survival (months)** |
| **iCCA_1** | 83 | 2 | 1 | 3 | yes | no | >32 | 2 |
| **iCCA_2** | 56 | 1 | 0 | 2 | yes | yes | 11 | 1 |
| **iCCA_3** | 66 | 2 | 0 | 3 | yes | yes | 22 | 1 |
| **iCCA_4** | 85 | 2 | - | 2 | no | no | >34 | >8 |
| **iCCA_5** | 63 | 2 | 1 | 2 | yes | yes | 11 | 4 |
| **iCCA_6** | 74 | 3 | 0 | 1 | no | no | >31 | >22 |
| **iCCA_7** | 38 | - | - | - | inoperable | yes | 3 | - |
| **iCCA_8** | 78 | 2 | 1 | 3 | no | yes (not cancer related) | 5 | 5 |
| **iCCA_9** | 67 | 1 | 0 | 2 | yes | yes | 28 | 20 |
| **iCCA_10** | 31 | - | - | - | inoperable | yes | 17 | - |
| **iCCA_11** | 59 | 1 | 0 | 2 | yes | no | >38 | 13 |
| **iCCA_12** | 61 | 1 | 0 | 2.5 | yes | no | >37 | 18 |
| **iCCA_13** | 53 | 2 | 1 | 2.5 | yes | yes | 36 | 26 |
| **iCCA_14** | 77 | 2 | - | 2 | yes | yes | 24 | 10 |
| **iCCA_15** | 56 | 2 | 1 | 3 | yes | no | >30 | 17 |
| **iCCA_16** | 76 | 2 | 1 | 3 | no | yes (not cancer related) | 3 | 3 |
| **iCCA_17** | 75 | 1 | 0 | 2.5 | no | No | >27 | >27 |
| T (tumor size) and N (positive lymph node) based on TNM classification, G (tumor differentiation grade) | | | | | | | | |

| **Supplemental Table 2: List of detected hydroxylated sulfatides** | | | | |  |
| --- | --- | --- | --- | --- | --- |
| Sulfatide | Total carbon atoms | Unsaturations | Neutral mass | *m/z* [M-H]^-^ | Tentative id |
| ST-OH | 30 | 1 | 739.4540 | 738.4468 | d18:1 C12:0h / d18:0 C12:1h |
| ST-OH | 32 | 1 | 767.4853 | 766.4781 | d18:1 C14:0h/d18:0 C14:1h |
| ST-OH | 33 | 1 | 781.5010 | 780.4937 | d18:1 C15:0h / d18:0 C15:1h |
| ST-OH | 34 | 0 | 797.5323 | 796.5250 | d18:0 C16:0h |
| ST-OH | 34 | 1 | 795.5166 | 794.5094 | **d18:1 C16:0h** |
| ST-OH | 34 | 2 | 793.5010 | 792.4937 | d18:1 C16:1h |
| ST-OH | 37 | 1 | 837.5636 | 836.5563 | d18:1 C19:0h / d18:0 C19:1h |
| ST-OH | 38 | 1 | 851.5792 | 850.5720 | d18:1 C20:0h / d18:0 C20:1h |
| ST-OH | 39 | 1 | 865.5949 | 864.5876 | d18:1 C21:0h / d18:0 C21:1h |
| ST-OH | 39 | 2 | 863.5792 | 862.5720 | d18:1 C21:1h |
| ST-OH | 40 | 0 | 881.6262 | 880.6189 | d18:0 C22:0h |
| ST-OH | 40 | 1 | 879.6105 | 878.6033 | **d18:1 C22:0h** |
| ST-OH | 40 | 2 | 877.5949 | 876.5876 | d18:1 C22:0h |
| ST-OH | 41 | 0 | 895.6418 | 894.6346 | d18:0 C23:0h |
| ST-OH | 41 | 1 | 893.6262 | 892.6189 | d18:1 C23:0h / d18:0 C23:1h |
| ST-OH | 41 | 2 | 891.6105 | 890.6033 | **d18:1 C23:1h** |
| ST-OH | 42 | 0 | 909.6575 | 908.6502 | d18:1 C24:0h / d18:0 C24:1h |
| ST-OH | 42 | 1 | 907.6418 | 906.6346 | **d18:1 C24:0h** |
| ST-OH | 42 | 2 | 905.6262 | 904.6189 | **d18:1 C24:1h** |
| ST-OH | 43 | 0 | 923.6731 | 922.6659 | d18:0 C25:0 |
| ST-OH | 43 | 1 | 921.6575 | 920.6502 | d18:1 C25:0h / d18:0 C25:1h |
| ST-OH | 43 | 2 | 919.6418 | 918.6346 | d18:1 C25:1h |
| ST-OH | 44 | 1 | 935.6731 | 934.6659 | **d18:1 C26:0h** |
| ST-OH | 44 | 2 | 933.6575 | 932.6502 | d18:1 C26:1h |

| **Supplemental Table 3: List of detected non-hydroxylated sulfatides** | | | | |  |
| --- | --- | --- | --- | --- | --- |
| Sulfatide | Total carbon atoms | Unsaturations | Neutral mass | *m/z* [M-H]^-^ | Tentative id |
| ST | 32 | 1 | 751.4904 | 750.4832 | d18:1 C14:0 / d18:0 C14:1 |
| ST | 33 | 0 | 767.5217 | 766.5145 | d18:1 C15:0 |
| ST | 34 | 0 | 781.5374 | 780.5301 | d18:1 C16:0 |
| ST | 34 | 2 | 777.5061 | 776.4988 | d18:1 C16:1 |
| ST | 37 | 0 | 823.5843 | 822.5771 | d18:0 C19:0 |
| ST | 38 | 1 | 835.5843 | 834.5771 | d18:1 C20:0 / d18:0 C20:1 |
| ST | 38 | 2 | 833.5687 | 832.5614 | d18:1 C20:1 |
| ST | 40 | 0 | 865.6313 | 864.6240 | d18:0 C22:0 |
| ST | 40 | 1 | 863.6156 | 862.6084 | **D18:1 C22:0** |
| ST | 40 | 2 | 861.6000 | 860.5927 | d18:1 C22:0 / d18:0 C22:1 |
| ST | 41 | 0 | 879.6469 | 878.6397 | d18:1 C14:0 / d18:0 C14:1 |
| ST | 41 | 1 | 877.6313 | 876.6240 | d18:1 C23:0 / d18:0 C23:1 |
| ST | 41 | 2 | 875.6156 | 874.6084 | d18:1 C23:1 |
| ST | 42 | 0 | 893.6626 | 892.6553 | d18:0 C24:0 |
| ST | 42 | 1 | 891.6469 | 890.6397 | **d18:1 C24:0 / d18:0 C24:1** |
| ST | 43 | 0 | 907.6782 | 906.6710 | d18:1 C25:0 |
| ST | 43 | 1 | 905.6626 | 904.6553 | d18:1 C25:0 / d18:0 C25:1 |
| ST | 43 | 2 | 903.6469 | 902.6397 | d18:1 C25:1 |
| ST | 44 | 0 | 921.6939 | 920.6866 | **d18:0 C26:0** |
| ST | 44 | 1 | 919.6782 | 918.6710 | d18:1 C26:0 / d18:0 C26:1 |
| ST | 44 | 2 | 917.6626 | 916.6553 | d18:1 C26:1 |

| **Supplemental Table 4. Top 10 of sulfatides with highest intensity in each group (median intensity).** | | | | | | |  |
| --- | --- | --- | --- | --- | --- | --- | --- |
| **Control** |  | **iCCA** |  | **CRLM** |  | **HCC** |  |
| ST-OH [34:1] | (4962) | ST-OH [42:1] | (15044) | ST-OH [42:1] | (272331) | ST-OH [37:1] | (5527) |
| ST-OH [39:2] | (4677) | ST-OH [42:2] | (3743) | ST-OH [34:1] | (186397) | ST-OH [39:2] | (1698) |
| ST-OH [42:1] | (4100) | ST [37:0] | (1730) | ST-OH [41:1] | (52758) | ST [33:0] | (1277) |
| ST-OH [40:1] | (1788) | ST-OH [40:1] | (1327) | ST-OH [42:0] | (52199) | ST-OH [39:1] | (1006) |
| ST-OH [41:1] | (1422) | ST-OH [34:0] | (825) | ST-OH [40:1] | (49917) | ST-OH [34:0] | (441) |
| ST-OH [34:0] | (887) | ST-OH [41:1] | (793) | ST-OH [34:0] | (21538) | ST [37:0] | (388) |
| ST-OH [42:2] | (541) | ST-OH [34:1] | (716) | ST [34:2] | (10511) | ST-OH [30:1] | (226) |
| ST [42:1] | (438) | ST-OH [40:2] | (647) | ST [34:0] | (10251) | ST-OH [34:1] | (151) |
| ST-OH [39:1] | (394) | ST [33:0] | (646) | ST-OH [41:0] | (9046) | ST-OH [41:2] | (110) |
| ST-OH [30:1] | (351) | ST [42:1] | (513) | ST [40:1] | (7108) | ST-OH [41:1] | (105) |

| **Supplemental Table 5. *P* values Cox Proportional Hazard analysis – iCCA patients** | | | |
| --- | --- | --- | --- |
| **features** | **disease-free survival** | **overall survival** | **disease-related survival** |
| Sum ST-OH | 0.594 | 0.765 | 0.961 |
| Sum ST | 0.934 | 0.697 | 0.967 |
| Sum total sulfatides | 0.816 | 0.717 | 0.991 |
| Sum Saturated ST-OH | 0.910 | 0.478 | 0.733 |
| Sum unsaturated ST-OH | 0.545 | 0.829 | 0.904 |
| Sum Saturated.ST | 0.882 | 0.597 | 0.807 |
| Sum unsaturated.ST | 0.398 | 0.920 | 0.544 |
| Hydroxylation ratio | 0.379 | 0.435 | 0.202 |
| Ratio unsaturated/saturation | **0.078** | **0.041** | 0.162 |
| Sum even-chain | 0.795 | 0.275 | 0.298 |
| Sum odd-chain | 0.948 | 0.309 | 0.322 |
| Ratio even-chain/odd-chain | 0.731 | 0.283 | 0.262 |

| **Supplemental Table 6. *P* values Cox Proportional Hazard analysis – CRLM patients** | | |
| --- | --- | --- |
| **features** | **disease-free survival** | **overall survival** |
| Sum ST-OH | 0.236 | 0.148 |
| Sum ST | 0.176 | 0.543 |
| Sum total sulfatides | 0.219 | 0.158 |
| Sum Saturated ST-OH | 0.181 | 0.503 |
| Sum unsaturated ST-OH | 0.262 | 0.139 |
| Sum Saturated.ST | 0.368 | 0.833 |
| Sum unsaturated.ST | 0.125 | 0.401 |
| Hydroxylation ratio | 0.366 | 0.478 |
| Ratio unsaturated/saturation | 0.433 | 0.948 |
| Sum even-chain | 0.227 | 0.162 |
| Sum odd-chain | 0.184 | 0.140 |
| Ratio even-chain/odd-chain | 0.274 | 0.928 |

| **Supplemental Table 7. *P* values Cox Proportional Hazard analysis – HCC patients** | | |
| --- | --- | --- |
| **features** | **disease-free survival** | **overall survival** |
| Sum ST-OH | 0.454 | 0.349 |
| Sum ST | 0.954 | 0.970 |
| Sum total sulfatides | 0.533 | 0.463 |
| Sum Saturated ST-OH | 0.390 | 0.521 |
| Sum unsaturated ST-OH | 0.400 | 0.322 |
| Sum Saturated.ST | 0.976 | 0.974 |
| Sum unsaturated.ST | 0.266 | 0.924 |
| Hydroxylation ratio | 0.668 | 0.768 |
| Ratio unsaturated/saturation | 0.385 | 0.637 |
| Sum even-chain | 0.550 | 0.672 |
| Sum odd-chain | 0.642 | 0.514 |
| Ratio even-chain/odd-chain | 0.855 | 0.195 |
